# Mechanism of metamifop inhibition of the carboxyltransferase domain of acetyl-coenzyme A carboxylase in *Echinochloa crus-galli*

**DOI:** 10.1038/srep34066

**Published:** 2016-09-26

**Authors:** Xiangdong Xia, Wenjie Tang, Shun He, Jing Kang, Hongju Ma, Jianhong Li

**Affiliations:** 1 Department of Plant Protection, College of Plant Science and Technology, Huazhong Agricultural University, Wuhan, Hubei, China; 2 Department of Applied Chemistry, College of Science, Huazhong Agricultural University, Wuhan, Hubei, China

## Abstract

Acetyl-coenzyme A carboxylase (ACCase) plays crucial roles in fatty acid metabolism and is an attractive target for herbicide discovery. Metamifop is a novel ACCase-inhibiting herbicide that can be applied to control sensitive weeds in paddy fields. In this study, the effects of metamifop on the chloroplasts, ACCase activity and carboxyltransferase (CT) domain gene expression in *Echinochloa crus-galli* were investigated. The results showed that metamifop interacted with the CT domain of ACCase in *E. crus-galli*. The three-dimensional structure of the CT domain of *E. crus-galli* ACCase in complex with metamifop was examined by homology modelling, molecular docking and molecular dynamics (MD) simulations. Metamifop has a different mechanism of inhibiting the CT domain compared with other ACCase inhibitors as it interacted with a different region in the active site of the CT domain. The protonation of nitrogen in the oxazole ring of metamifop plays a crucial role in the interaction between metamifop and the CT domain. The binding mode of metamifop provides a foundation for elucidating the molecular mechanism of target resistance and cross-resistance among ACCase herbicides, and for designing and optimizing ACCase inhibitors.

Metamifop (CAS number: 256412-89-2), developed by Dongbu Hannong Chemical Co., Ltd. (Seoul, Korea), is a postemergence herbicide classified as a lipid synthesis inhibitor (inhibition of acetyl-coenzyme A carboxylase (ACCase)) according to the Herbicide Resistance Action Committee (HRAC; http://www.hracglobal.com). Metamifop exhibits high control efficacy against sensitive weeds, especially *Echinochloa crus-galli* in paddy fields. ACCase plays a crucial role in fatty acid biosynthesis in plants and is an attractive target for herbicide exploitation^1^. ACCase herbicides have been widely used for weed control for over 25 years^2^. Currently, 47 biotypes of ACCase-inhibition herbicide-resistant weeds have been reported^3^, causing differing degrees of failure in weed control.

ACCase, a biotin-dependent enzyme, catalyses the formation of malonyl-CoA from the ATP-dependent carboxylation of acetyl-CoA^4^. Two isoforms of ACCases have been identified: one is heteromeric ACCase (multiple subunits) and the other is homomeric ACCase (a single polypeptide). The heteromeric ACCase, which occurs in the plastids of plant (except for monocots) and prokaryotic cells^5^,^6^, is not sensitive to ACCase inhibitors. In contrast, the homomeric ACCase, which occurs widely in the cytosol of plants, mammals, other animals, yeasts, fungi and the plastids of monocots, is sensitive to ACCase-inhibiting herbicides^7^. Thus, the plastid ACCase in monocots is the target enzyme for ACCase herbicides, whereas most dicots tolerate the herbicides well, indicating that ACCase herbicides are selective between monocots and dicots^7^. In addition, some cereal crops metabolize the herbicides to inactive compounds, providing good selectivity between weeds and the crops^8^.

ACCase-inhibiting herbicides, such as haloxyfop, diclofop, and sethoxydim, inhibit the CT domain of ACCase^9^,^10^ and result in the inhibition of lipid metabolism^11^, eventually killing the plants. The crystal structures of the *Staphylococcus aureus* (yeast) CT domain in complex with haloxyfop^12^, tepraloxydim^13^, and pinoxaden^14^ showed that these herbicides occupied the active site by competing with acetyl-CoA and were located at different regions of the dimer interface of the CT domain. Although these three different classes of ACCase herbicides have diverse chemical structures, they share two common anchoring points (Ile1735 and Ala1627) with the yeast CT domain^12^,^13^,^14^. Therefore, these results confirmed that the two sites played a key role in binding with these herbicides. Computational simulations of the *Setaria italica* CT domain in complex with ACCase inhibitors suggested that the orientation of the carboxyl group of the inhibitors bound to the binding pocket differed, but these ACCase inhibitors can form a hydrogen-bond with the Ser698 residue^15^. These results indicated that there are many different molecular mechanisms for inhibiting the CT domain, but these compounds may commonly bind with the CT domain.

Although the binding mode of herbicides to the CT domain of yeast ACCase was previously established, these herbicides exhibited low inhibitory activity against the yeast CT domain. Some residues in the active region of the dimer interface between the plant and other ACCases exhibited significant variations that may lead to a change in the dimer organization for herbicide binding^12^. It is likely that the binding mode of herbicides to ACCase from yeast cannot completely represent the binding mode of sensitive plants. Metamifop is a novel ACCase-inhibition herbicide and its detailed molecular mechanism has not been reported. In this study, the effects of metamifop on the chloroplasts, ACCase activity and CT domain gene expression in *E. crus-galli* are described. The structure of the CT domain of *E. crus-galli* ACCase in complex with metamifop was examined by homology modelling and molecular dynamics (MD) simulations. The mechanism of CT domain inhibition by metamifop was rather different from that of other ACCase herbicides. This study provides a foundation for elucidating the molecular mechanism of target resistance and cross-resistance among ACCase herbicides, and for designing and optimizing ACCase inhibitors. Moreover, molecular insight into the interactions between metamifop and the CT domain may aid in understanding the significance of the protonation of compounds.

## Results

### Metamifop causes ultrastructural damage to chloroplasts

**Figure 1:**
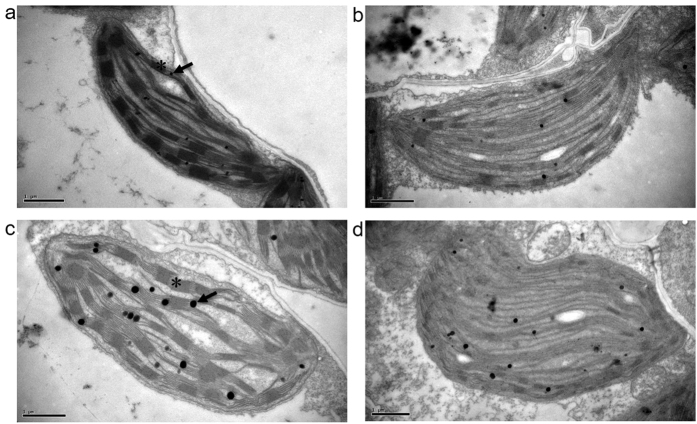
TEM observation of chloroplasts (3 DAT). Electron microscopy images of chloroplasts from *Echinochloa crus-galli* grown under control conditions (**a**) and with different doses of metamifop treatments (**b-d**: 12.5, 45, and 90 g a.i. ha^−1^) are presented. With increasing doses of metamifop treatment, the ultrastructure was more severely destroyed. Arrow: plastoglobule, asterisk: thylakoid. Bars: 1 μm.

To investigate the effect of metamifop on the physical organization of chloroplast in plant cells, the ultrastructure was examined by transmission electron microscopy (TEM). Significant alteration in the chloroplast structure was observed in metamifop-sprayed plants compared with healthy control plants. Electron micrographs of chloroplasts from metamifop-sprayed plants, compared with control plants (Fig. 1a) at the same growth stage, showed partial disappearance of the thylakoid structure and loose stacking of the grana (Fig. 1b,c). A high dose of metamifop caused the almost complete disappearance of thylakoids, disrupted stacking of grana and led to an irregular shaped chloroplasts (Fig. 1d), which confirmed the role of metamifop in damaging chloroplasts. In addition, increased amounts of plastoglobules were noted in the chloroplasts of plants treated with a relatively high dose of metamifop (Fig. 1c,d) compared with the control and plants treated with a low dose of metamifop (Fig. 1a,b). Metamifop caused more severe damage to the chloroplasts, and the degree of damage was increased with increasing doses.

### CT domain of chloroplastic ACCase from *E. crus-galli*

**Figure 2:**
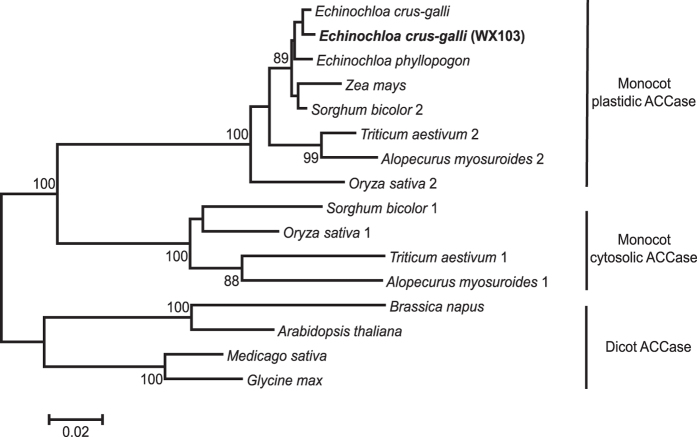
Phylogenetic tree from analysis of ACCase by the neighbour-joining method. The accession numbers are as follows: *Echinochloa crus-galli* (HQ395758), *Echinochloa phyllopogon* (BAL49670), *Zea mays* (U19183), *Sorghum bicolor* 1 and 2 (XM_002442197 and XM_002446133), *Triticum aestivum* 1 and 2 (U39321 and AF029895), *Alopecurus myosuroides* 1 and 2 (AJ632096 and AJ310767), *Oryza sativa* 1 and 2 (Os10g21910 and Os05g22940), *Brassica napus* (AJ131865), *Arabidopsis thaliana* (L27074), *Medicago sativa* (L25042), and *Glycine max* (XM_003522763). *Echinochloa crus-galli* (WX103 biotype) was cloned. Its accession number is KU198448, and it clustered to the monocot plastidic ACCase.

The length of the CT domain of ACCase in *E. crus-galli* is 1659 bp (accession number KU198448), encoding 553 amino acid residues corresponding to residues 1640 to 2193 of *Alopecurus myosuroides* (accession number AJ310767). The deduced amino acid sequence exhibited approximately 93% identity with the CT domain of plastidic ACCase from *A. myosuroides*, whereas only 78% amino acid identity was observed with cytosolic ACCase from *A. myosuroides* (accession number AJ632096). A phylogenetic tree of the CT domain in plant ACCase showed that the CT domain gene cloned from sensitive *E. crus-galli* was classified in the plastidic clades (Fig. 2). This result revealed that the isolated CT domain gene encoded the CT domain of chloroplastic ACCase. Generally, single site mutations or multiple mutants of seven amino acid sites (Ile1781, Trp1999, Trp2027, Val2041, Asp2078, Cys2088 and Gly2096) in the plant CT domain of chloroplastic ACCase confer ACCase inhibitor resistance^2^. The CT domain of chloroplastic ACCase from sensitive *E. crus-galli* includes these seven amino acid sites without mutation. This finding confirmed again that the isolated CT domain gene encodes the chloroplastic ACCase in *E. crus-galli*.

### Metamifop affects CT domain gene expression

**Figure 3:**
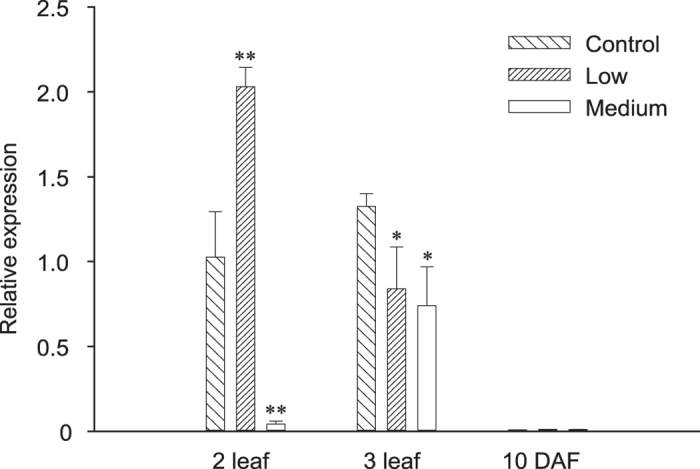
CT domain gene expression in treated and untreated plants. The expression of the CT domain gene at the 2-leaf, 3-leaf and 10 DAF stages was quantified relative to the β-actin gene with qRT-PCR. The expression of the CT domain gene in plants treated with a low dose of metamifop (left diagonal filled bars), plants treated with a medium dose of metamifop (white colour filled bars), and untreated plants (right diagonal filled bars) is normalized to the expression of the gene in untreated plants at the 2-leaf stage. Error bars indicate standard deviation. **p < 0.01 and *p < 0.05 indicate significant differences relative to untreated plants at each stage.

The expression of the CT domain gene in *E. crus-galli* was investigated using qRT-PCR (Fig. 3). Significant differences in the expression of the CT domain gene were detected in metamifop-treated plants compared with healthy control plants at early development stages (2-leaf and 3-leaf stages). At a late growth stage (10 days after flowering, DAF), no remarkable changes concerning the expression of the CT domain gene were observed among the different doses of metamifop-treated plants and non-treated plants. It was clear that the expression of the CT domain gene was extremely low in healthy control plants and in metamifop-treated plants at the 10 DAF stage.

The peak expression level of the CT domain gene from plants treated with a low dose of metamifop (L; 12.5 g a.i. ha^−1^) at the 2-leaf stage (1 day after treatment, DAT) was increased approximately 2.0-fold compared with healthy control plants. In contrast, reduced expression was found at the 2-leaf stage in plants sprayed with a medium dose of metamifop (M; 45.0 g a.i. ha^−1^) compared with control plants. As the plants grew to the 3-leaf stage, slightly significant differences in the expression of the CT domain gene were noted between metamifop-treated and healthy control plants. Interestingly, the expression of the CT domain was significantly upregulated from the 2-leaf stage to the 3-leaf stage at a medium dose of metamifop.

### Inhibition of ACCase activity by metamifop

**Figure 4:**
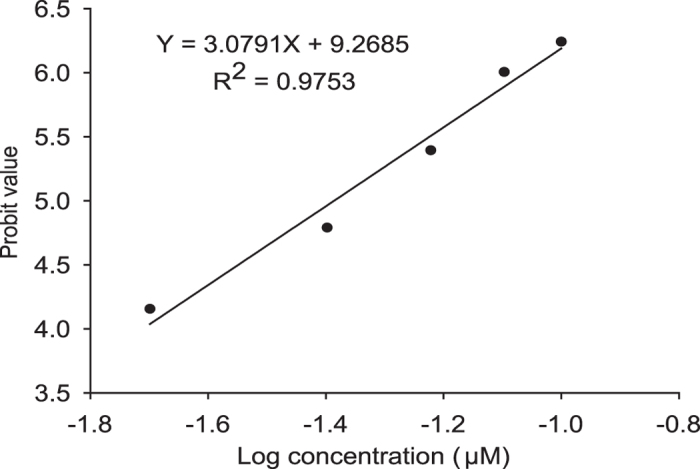
Inhibition of ACCase activity by metamifop. The linear model was fitted by plotting probit values of the average ACCase activity inhibition rate against the log dose of metamifop. IC_50_ = 41.1 nM.

The effect of different doses of metamifop on ACCase activity was determined using a dose-response curve (Fig. 4). The triplicate results of ACCase activity revealed no significant differences at the same dose, so the average value was used to calculate the probit value of the inhibition rate. In this study, the 50% inhibitory concentration (IC_50_) value for metamifop was 41.1 nM. These results verified that metamifop efficiently inhibited ACCase activity.

### Mechanism of the inhibition of the CT domain in *E. crus-galli* by metamifop

The sequence identity between the CT domain of *E. crus-galli* ACCase and yeast ACCase was 53%; thus, the information from the crystal structure of the yeast CT domain (PDB code: 1UYS and 1UYT) should be applied to homology modelling of the *E. crus-galli* CT domain^16^. The quality of the homology model of the *E. crus-galli* CT domain was good, with most of the residues (91.5%) in the most favoured region of the Ramachandran plot and only 0.2% in the disallowed region (Supplemental Fig. S1). The CT domain was a homodimer formed by the side-to-side arrangement of two monomers in such a manner that the N domain of one molecule was positioned next to the C domain of the other. The molecular conformations of metamifop were docked into the active site of the CT domain by the Surflex program. Interestingly, the molecular docking results showed that metamifop with protonation of the nitrogen on the oxazole ring in complex with the CT domain presented a reasonable conformation with the best scores.

**Figure 5:**
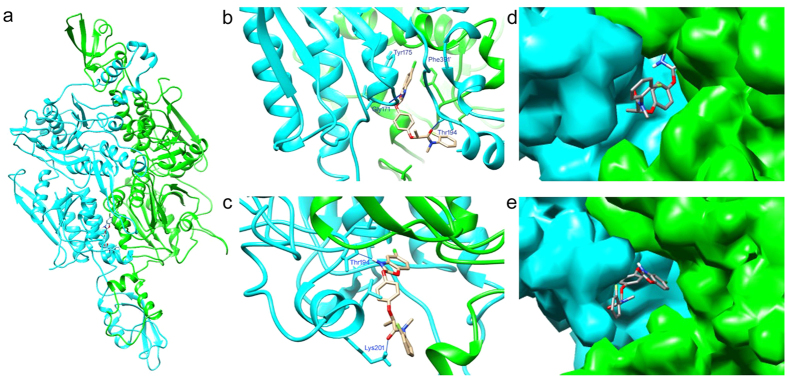
Binding of metamifop in the active site of the CT domain. (**a**) Binding of metamifop in the active site of the CT domain (cyan and green for two monomers) in *Echinochloa crus-galli* ACCase. Metamifop is presented as a stick diagram. (**b**) Stereo drawing of the active site of the CT domain in complex with metamifop. (**c**) Stereo drawing of metamifop moving to the entrance of the binding channel of the CT domain. (**d**) Molecular surface of the binding pocket of CT in complex with metamifop. (**e**) Molecular surface of the entrance of the binding channel of the CT domain in complex with metamifop. Hydrogen bonds are indicated with blue lines. Drawings were made using UCSF Chimera^47^.

To obtain a more reasonable and stable structure of the *E. crus-galli* CT domain in complex with metamifop, MD simulations were performed. The root-mean-square deviation (RMSD) plot of the Cα atoms for the CT domain and the total energy (ETOT) plot for the simulation system indicated that the system had achieved equilibrium after relaxation (Supplemental Fig. S2). In this study, metamifop was bound in the active site of the CT domain at its dimer interface (Fig. 5a). In the first stage, metamifop was bound in the binding pocket of the CT dimer interface, and the average structure was extracted from 10 to 380 ps (Fig. 5b,d). The oxazole ring of metamifop was sandwiched between the side chains of Tyr175 and Phe391’ (corresponding to Tyr1738 and Phe1956’ in the yeast CT domain, the residue numbers with primes indicate the other monomer), showing π-π interactions. More importantly, the protonation of nitrogen on the oxazole ring of metamifop played a key role in the interactions between metamifop and the CT domain. The additional proton gained by protonation was hydrogen-bonded to the main-chain amide of Gly171 (corresponding to Gly1734 next to Ile1735 in the yeast CT domain), and the oxygen atom on the amide was hydrogen-bonded to Thr194.

The residue Thr194 played a key role in the interaction with metamifop. At the first stage, the oxygen on amide was hydrogen-bonded to Thr194 (Fig. 5b). At the second stage, a large conformational change in Tyr175 and Phe391’ was observed at 382 ps. The two residues behaved as a gate. Metamifop moved from the binding pocket of the CT domain to the entrance of the binding channel (Fig. 5e) when the gate closed. The structure showed that the additional proton gained by protonation of nitrogen on the oxazole ring of metamifop was hydrogen-bonded to Thr194 and the oxygen atom on the amide was hydrogen-bonded to Lys201 (Fig. 5c).

**Figure 6:**
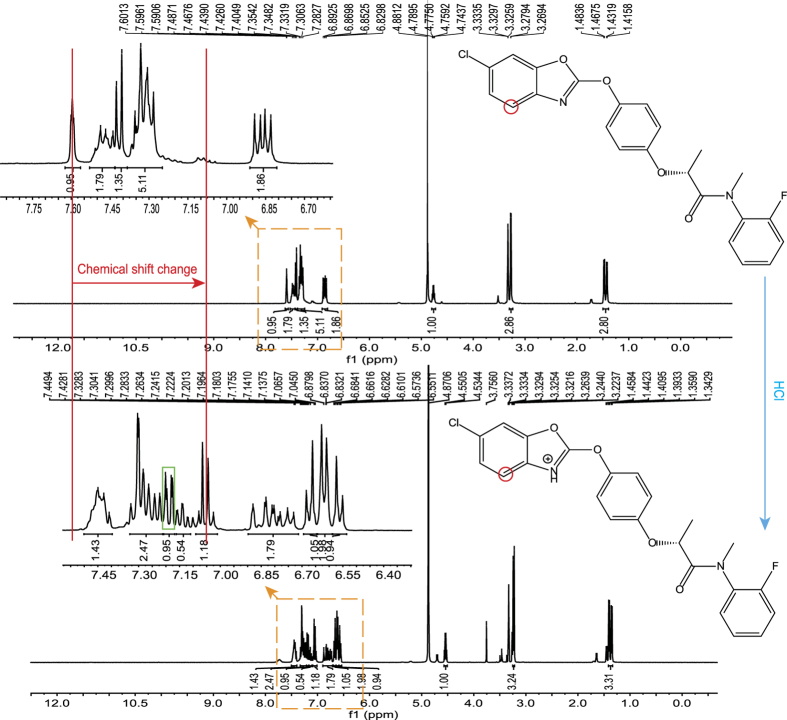
Protonation of nitrogen in oxazole ring of metamifop. *In vitro*, the reaction was conducted under acidic conditions. After the reaction, a significant change in the chemical shift (indicated by red arrow) of the hydrogen (indicated by red circle) was observed in the ^1^H NMR spectrum diagrams. Moreover, an additional proton (indicated by green square) was also detected in the diagram.

The energy decomposition of the binding free energy suggested that glutamates (Glu400’, Glu411’, Glu429’, and Glu461’) and aspartates (Asp363’, Asp397’, and Asp439’) were key residues for electrostatic interactions. These acidic amino acids generated an acidic environment for metamifop interactions with the active site of the dimer interface. However, the phenomenon of protonation of nitrogen on the oxazole ring in metamifop could not occur when these acidic amino acids were mutated to neutral or basic amino acid residues; thus, metamifop could not form a hydrogen-bond to link Thr194. More importantly, the binding free energy of metamifop in complex with the mutant CT domain was significantly increased compared with the wild type CT domain, indicating that metamifop cannot bind with mutated CT domain well. Based on the theory of the computational results, we hypothesized that the protonation of nitrogen in the oxazole ring of metamifop can occur spontaneously under acidic conditions *in vitro*. Therefore, an experiment of protonation of metamifop *in vitro* was performed as described in the Methods. The ^1^H NMR spectrum diagrams of metamifop, protonated metamifop (metamifopH), and propanamide (Supplemental Figs S3–5) were analysed. The results revealed that the chemical shift of the hydrogen closest to the nitrogen in the oxazole ring changed from 7.60 to 7.05 ppm after reaction (Fig. 6). Moreover, an additional proton was detected in the ^1^H NMR spectrum diagram of metamifopH (Fig. 6). The results demonstrated that protonation of the nitrogen occurred in acidic conditions and further confirmed that the structure of the *E. crus-galli* CT domain in complex with metamifop was reasonable.

## Discussion

The molecular mechanism for the inhibitory action of metamifop has not been previously reported. In this study, we clearly demonstrated that metamifop was bound in the active site of the CT domain of chloroplast ACCase in *E. crus-galli* and inhibited the CT domain activity of ACCase, thereby shutting down fatty acid biosynthesis. Metamifop may imitate the behaviour of the substrate biotin to protonate the nitrogen of the oxazole ring, form an NH- functional group and then interact with the CT domain.

Metamifop caused more severe damage to the ultrastructure of chloroplasts with increased doses. Increased amounts of plastoglobules in the chloroplasts of plants were noted with a relatively high dose of metamifop (Fig. 1c,d). Plastoglobules were chloroplast lipid droplets attached to the thylakoid membranes^17^,^18^, containing a high level of α-tocopherol that protected thylakoid membranes from photoinhibition and oxidative stress^17^. The number of plastoglobules in the chloroplast was observed to increase under drought^18^ and nitrogen starvation^19^. In addition, plastoglobules not only stored lipids but also actively participated in lipid metabolism^20^. Hence, the increasing amount of plastoglobules may be a stress response of plants to metamifop-influenced lipid metabolism.

ACCase played a crucial role in the lipid metabolism in plants; therefore, it was an attractive target for herbicide discovery^1^. ACCase herbicides including haloxyfop, tepraloxydim, and pinoxaden, killed sensitive plants by inhibiting CT domain activity in chloroplast ACCase^7^,^12^,^13^,^14^. In this study, significant differences in the expression of the CT domain gene in metamifop-treated plants compared with healthy control plants were detected at early development stages. Interestingly, the expression of the CT domain was significantly upregulated from the 2-leaf stage to the 3-leaf stage with a medium dose of metamifop (Fig. 3). Upregulation of the expression level of ACCase gene may be related to stress, resulting in increasing concentrations of malonyl-CoA for fatty acid synthesis^21^. The results indicated that metamifop, regarded as a stress, played an important role in altering the expression of CT domain of chloroplast ACCase in *E. crus-galli*. Additionally, the effect of different doses of metamifop on ACCase activity was determined using a dose-response curve, which demonstrated that metamifop can efficiently inhibit ACCase activity. A previous study also found the IC_50_ value for quizalofop-P-ethyl (an ACCase inhibitor) for the inhibition of *E. crus-galli* ACCase (Geqiushan S biotype) was 41 nM^22^. Metamifop exhibited the same level of activity for inhibition of plant ACCases as quizalofop-P-ethyl. The results of the TEM observation, CT domain gene expression and ACCase activity assay indicated that metamifop was very likely interacting with the CT domain of chloroplast ACCase in *E. crus-galli*.

To decipher the molecular mechanism of metamifop inhibition of the CT domain of acetyl-coenzyme A carboxylase in *E. crus-galli*, the three-dimensional structure of CT domain of *E. crus-galli* ACCase in complex with metamifop was formed and examined by homology modelling, molecular docking and MD simulations. The structure clearly showed that metamifop was bound in the active site of the CT domain of *E. crus-galli* ACCase at its dimer interface. However, metamifop interacted with a different region from other ACCase inhibitors, as represented by haloxyfop^12^, tepraloxydim^13^, and pinoxaden^14^. In the first stage, metamifop was bound in the binding pocket of the CT dimer interface. Tyr175 and Phe391’ of the CT domain interacted with the oxazole ring in metamifop via π-π interactions. These key interactions were similar to the crystal structure of the yeast CT domain in complex with haloxyfop and diclofop^12^. Computational simulations of the structure of the *A. myosuroides* CT domain in complex with haloxyfop and clodinafop also revealed these key interactions^23^.

More importantly, the protonation of nitrogen on the oxazole ring of metamifop played a key role in binding interactions between metamifop and the CT domain. The additional proton gained by protonation was hydrogen-bonded to Gly171 (corresponding to Gly1734 next to Ile1735 in the yeast CT domain). Similarly, the crystal structure of the sixth CT domain of *Mycobacterium tuberculosis* ACCase in complex with haloxyfop revealed that Gly138 (corresponding to Gly171) hydrogen-bonded with the carboxylate oxygen of haloxyfop^24^. Haloxyfop, tepraloxydim and pinoxaden interacted with various regions of the CT domain, sharing two common points (Ile1735 and Ala1627) of interaction with the yeast CT domain^12^,^13^,^14^. Some residues in the region of the dimer interface from plant ACCases and yeast ACCase exhibited significant variations that may lead to a change in dimer organization for herbicide binding^12^. In addition, conformational changes in the active site region were crucial for the formation of the inhibitor binding site^12^,^13^,^14^. Therefore, the residue Gly171 of the CT domain of ACCase in *E. crus-galli* was crucial for binding with metamifop.

The active site Ile172 that is equivalent to Ile1781 in the CT domain of *A. myosuroides* may play a crucial role in ACCase-inhibition herbicides binding to the CT domain of grass ACCase. Gramineous weeds resistant to ACCase-inhibition herbicides have increased rapidly, mostly caused by a point mutation or multiple mutations in the CT domain of chloroplastic ACCase. Interestingly, the Ile1781/Leu mutation has been identified in almost all resistant weeds^25^,^26^. However, the ACCase of *Toxoplasma gondii* had a Leu residue at position 1705 (allele to 1781 in *A. myosuroides*) but was still sensitive to the aryloxyphenoxypropionate herbicides^27^. Therefore, herbicide sensitivity may not be solely determined by the residue Ile172.

The residue Thr194 also played a key role in the interaction with metamifop. Initially, the oxygen on amide was hydrogen-bonded to Thr194 (Fig. 5b). However, a large conformational change was observed at 382 ps. When the residues Tyr175 and Phe391’ were closed similar to a gate, metamifop would gradually move from the binding pocket of the CT domain to the entrance of the binding channel. Then, Thr194 was hydrogen-bonded to the additional proton gained by protonation of metamifop. The structure of yeast CT domain in complex with haloxyfop or diclofop was unlikely to be stable. The formation of the binding site requires conformational variability for several residues in the active site of the enzyme. Factors that regulate the conformational dynamics of residues in this dimer interface may affect the inhibitor sensitivity of the CT domain^12^. In this study, the protonation of nitrogen on the oxazole ring of metamifop occurred spontaneously. The reaction released a large amount of heat, which caused a large conformational change in Tyr175 and Phe391’ and resulted in disturbing the π-π interaction. Therefore, metamifop moved from the binding pocket to the entrance of the binding channel, where it bound with Thr194 and Lys201 (Fig. 5c).

Metamifop imitated the behaviour of the substrate biotin to protonate the nitrogen of the oxazole ring and form an NH- functional group that was hydrogen-bonded to the CT domain. The crystal structure of *M. tuberculosis* CT domain in complex with haloxyfop showed that the side chain oxygen atom of Tyr326 (similar to Phe1956’ in the yeast structure) was hydrogen-bonded to the backbone nitrogen of Thr160 through a water linkage, making the binding pocket stable^15^. The space position of Thr194 in *E. crus-galli* was consistent with the position of Thr160 in *M. tuberculosis* AccD6, indicating that Thr194 may stabilize the interaction of CT domain with metamifop. The crystallographic structure of *Staphylococcus aureus* pyruvate carboxylase in complex with coenzyme A suggested that Thr908 was hydrogen-bonded to the N1′ atom of biotin. Importantly, a general acid was needed to protonate the N1′ atom of biotin^28^. Based on the theory of computational results, we hypothesized that an acidic environment was crucial for the protonation of the nitrogen on oxazole ring. *In vitro*, the protonation of the nitrogen on the oxazole ring was confirmed by our experiments. These results demonstrated that the protonation of the nitrogen would occur in the binding with the *E. crus-galli* CT domain.

Deciphering the mechanism of metamifop provides a foundation for elucidating the molecular basis of target resistance and cross-resistance among ACCase herbicides, and for designing and optimizing ACCase inhibitors against weeds. Moreover, this study may aid in understanding of the significance of the protonation of compound in interaction of compound with target enzyme.

## Methods

### Plant materials

*E. crus-galli* seeds collected from an untilled field in Wuxue County, China in 2011 were self-crossed for three successive generations as a susceptible biotype (WX103). WX103 is susceptible to metamifop, bispyribac-sodium, penoxsulam and quinclorac. Seeds of WX103 were germinated on wet paper for 48 h at 28 °C under constant light (approx. 3000 lx). The seedlings were transplanted to pots filled with a mixture of sand and loam in a greenhouse at 25 °C with a 16-h light/8-h dark photoperiod.

### Transmission electron microscopy

*E. crus-galli* plants grew to the 2-leaf stage and were treated with different doses of metamifop at 0, 12.5, 45 and 90 g a.i. ha^−1^. After three days, fresh leaves were harvested with a sharp razor blade and cut into small pieces. Then, the samples were fixed in 2.5% (v/v) glutaraldehyde and 1% (v/v) osmium tetroxide. The sections were treated as described by Wang, Y. *et al.*^29^. Microscopic observation was performed using an H-7650 transmission electron microscope (Hitachi, Tokyo, Japan).

### Sequencing of chloroplastic ACCase CT domain

Total RNA was isolated from leaves of *E. crus-galli* using the RNAprep Pure Plant Kit (Tiangen Biotech Co., Ltd., Beijing, China). For RT-PCR, first-strand cDNA synthesis was reverse transcribed from the total RNA using the RevertAid RT Kit (Thermo Scientific). The amplification product obtained from at least 6 independent PCR reactions was purified and then sequenced on both strands using gene-specific primers. The isolated CT domain sequence was aligned with other plant CT domain sequences from cytosol and plastid ACCases, and then a phylogenetic tree was constructed by the neighbour-joining method using Mega 6^30^. (See detailed procedure in supporting information.)

### Real-time PCR

The level of CT domain gene expression was analysed by real-time PCR using β-actin as the reference gene. Triplicate cDNA aliquots for each sample from individual plants were amplified by qPCR. The relative changes in gene expression were calculated by the 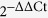 method^31^. Each experiment was repeated thrice. (See detailed procedure in supporting information).

### Inhibition of *E. crus-galli* ACCase activity by metamifop

When plants grew to the 2- to 3-leaf stage, the shoots were cut at the base and then stored at −80 °C. ACCase isolation was performed according to the procedures described by Cocker *et al.*^32^. The total protein contents of the ACCase isolations were determined as described by Bradford^33^.

The enzyme activity assay method was modified based on Zhibo H. *et al.*^22^ (see detailed ACCase activity assay method in supporting information). Biological triplicates of the assays were statistically analysed by ANOVA with Fisher’s Protected LSD test. The enzyme activities for the same treatment were averaged when there were no significant differences among three biological replicates. The data were fitted to the linear model by plotting the probit values of the average ACCase activity inhibition rate against the log dose of metamifop.

### Homology modelling and molecular docking

The amino acid sequence of *E. crus-galli* ACCase (accession number KU198448) was numbered 1 to 553. Based on the templates of the B and C chains from the crystal structures of the yeast CT domain and its complexes with haloxyfop and CoA (PDB code: 1UYS and 1UYT), the theoretical structure of the CT domain from *E. crus-galli* was developed using Modeller 9.12^34^. The quality of modelling was assessed by submitting the model to the online server http://services.mbi.ucla.edu/SAVES/. Finally, the most suitable model was selected for molecular docking with metamifop to build the theoretical model of the *E. crus-galli* CT domain in complex with metamifop (CT-metamifop complex) using the Surflex-docking program with Sybyl 7.3 (Tripos Inc., St Louis, MO, USA)^35^.

### Molecular dynamics simulations of the *E. crus-galli* CT domain in complex with metamifop

The CT-metamifop complex was subjected to MD simulations using the SANDER module of the AMBER 14 package^36^ to obtain a stable conformation. The antechamber module was used to prepare metamifop by the AM1-BCC method^37^,^38^. The pdb4amber program was applied to remove hydrogen atoms from the CT domain, and the program Reduce was used to add hydrogens^39^. The AMBER ff14SB force field^40^ and GAFF force field^41^ were adopted for the CT domain and metamifop, respectively. The complex was solvated in an octahedral box of TIP3P water molecules^42^, which extended 10 Å from any given atoms in the system, and then neutralized by adding Na^+^ ions.

The system was minimized in 2000 steps steepest descent (SD) and 2000 steps conjugate gradient (CG) with the system free and heated gradually from 0 to 300 K using Langevin thermostats with a collision frequency of 1.0 in 50 ps with the force constant of a restraint on all CA, C, N atoms of the complex at 4 kcal·mol^−1^·Å^2^. Then, the model was relaxed in 4 discrete simulation steps of 250 ps at 300 K and constant pressure (1 atm) by decreasing the force constant of the imposed position restraint on all CA, C, N atoms of the complex from 3 to 0 kcal·mol^−1^·Å^2^. The MD simulations ran for an additional 3 ns after relaxation at a constant temperature of 300 K with a time constant for heat bath coupling of 5 ps using the weak-coupling method. The Particle Mesh Ewald method^43^ was selected for periodic long-range electrostatic force and a 10 Å cutoff for nonbonding Van der Waals interactions. All bonds were constrained using the SHAKE algorithm^44^. The leap-frog algorithm^45^ was used with a timestep of 2 fs. Coordinates were recorded every 2 ps during the MD simulations.

### Binding free energy calculation (MM/GBSA)

Binding free energy and energy decomposition were calculated using the molecular mechanics-Generalized Born Surface Area (MM/GBSA) method^46^ by MMPBSA.py with the &gb and &decomp control procedure in Amber 14, respectively. Based on energy decomposition, the Van der Waals interactions between metamifop and each residue in the active pocket of the CT dimer were calculated.

For the first stage, metamifop binding at the active site of the CT dimer interface, structures were sampled from the MD trajectories from 10 to 380 ps. For the second stage, metamifop moving to the entrance of binding channel, CT-metamifop complexes were selected from 382 to 3000 ps for the binding free energy calculation.

### Protonation of nitrogen in oxazole ring of metamifop

*In vitro*, 0.1 M HCl was added to a solution of metamifop in MeOH, and the reaction system was agitated at room temperature for 12 h. The reaction products were neutralized with 0.1 M NaOH, and the target product was extracted by ethyl acetate. The solvent was removed in vacuo, and the product was dried by vacuum drier at 50 °C. The change in the chemical shift of the hydrogen closest to the nitrogen in the oxazole ring of metamifop, determined by comparing ^1^H NMR spectrum diagrams of the product to the spectrum of metamifop, was used to confirm the protonation of the nitrogen.

## Additional Information

**How to cite this article**: Xia, X. *et al.* Mechanism of metamifop inhibition of the carboxyltransferase domain of acetyl-coenzyme A carboxylase in *Echinochloa crus-galli. Sci. Rep.* **6**, 34066; doi: 10.1038/srep34066 (2016).

## Supplementary Material

Supplementary Information
