# Supplementary material for: Mechanism of metamifop inhibition of the carboxyltransferase domain of acetyl-coenzyme A carboxylase in Echinochloa crus-galli
Source: Sci Rep. 2016 Sep 26;6:34066. doi: 10.1038/srep34066 (PMC5036181; doi:10.1038/srep34066)
Supplement: Supplementary Information [file srep34066-s1.pdf]

Supporting information

Mechanism of metamifop inhibition of the carboxyltransferase domain of acetyl-coenzyme A carboxylase in *Echinochloa crus-galli*

Xiangdong Xia[1], Wenjie Tang[2], Shun He[1], Jing Kang[2], Hongju Ma[1]*, Jianhong Li[1]*

1 Department of Plant Protection, College of Plant Science and Technology, Huazhong Agricultural University, Wuhan, Hubei, China;

2 Department of Applied Chemistry, College of Science, Huazhong Agricultural University, Wuhan, Hubei, China;

* To whom correspondence should be addressed.

Hongju Ma E-mail: mahongju@mail.hzau.edu.cn;

Jianhong Li E-mail: jianhl@mail.hzau.edu.cn

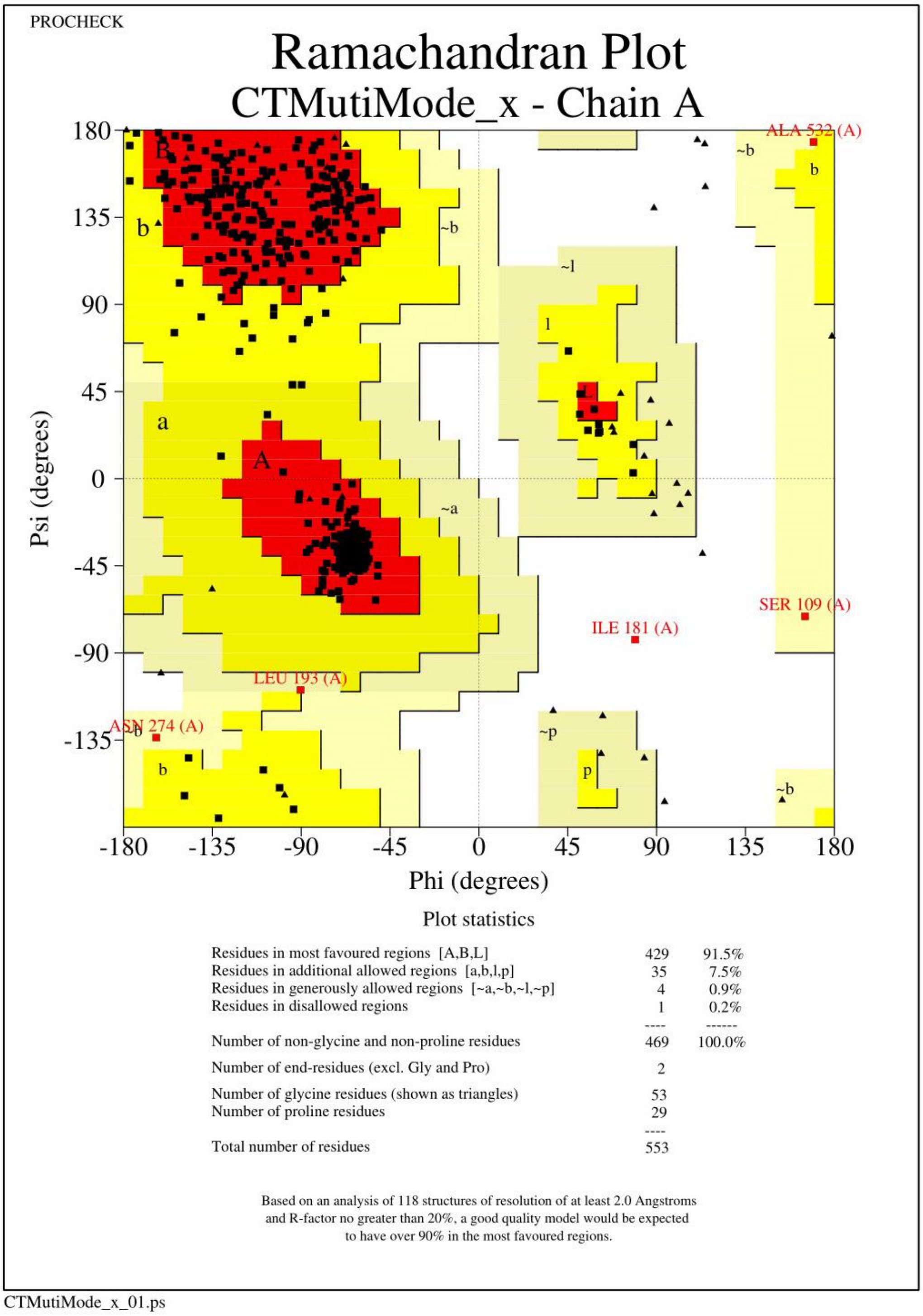

Figure S1. Ramachandran plot of CT domain of ACCase in *Echinochloa crus-galli*. The quality of the homology model of the barnyard grass CT domain was good, with most of the residues (91.5%) in the most favored region and 0.2% in the disallowed region.

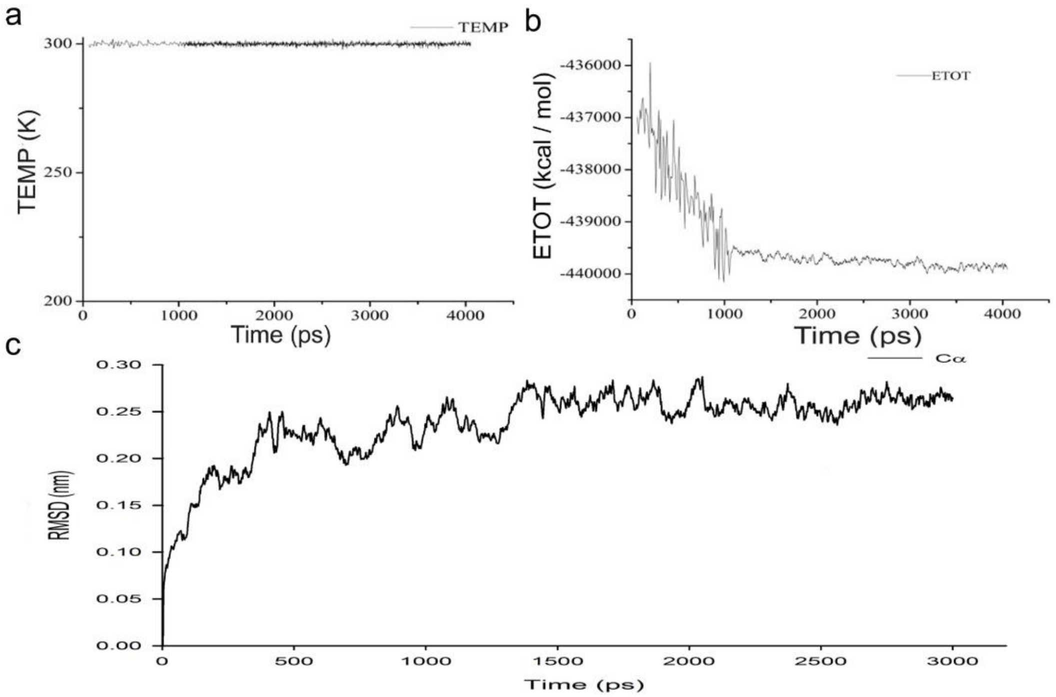

Figure S2. Equilibrium of the simulation system. (a) Plots of TEMP for the simulation system. (b) Plots of ETOT for the simulation system. (c) Plots of the RMSDs of Cα atoms for the CT domain.

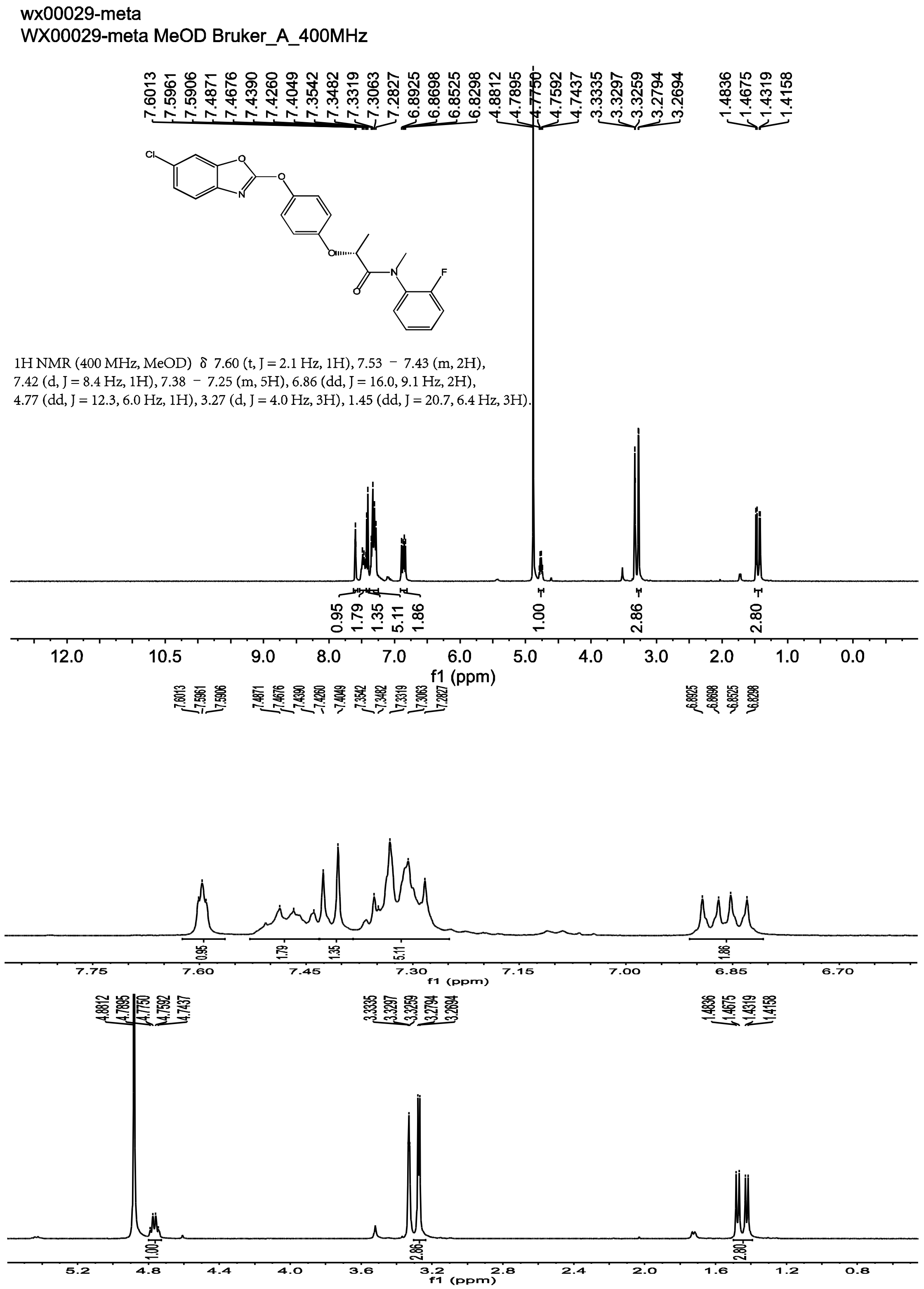

Figure S3. [1]**H NMR of metamifop.**

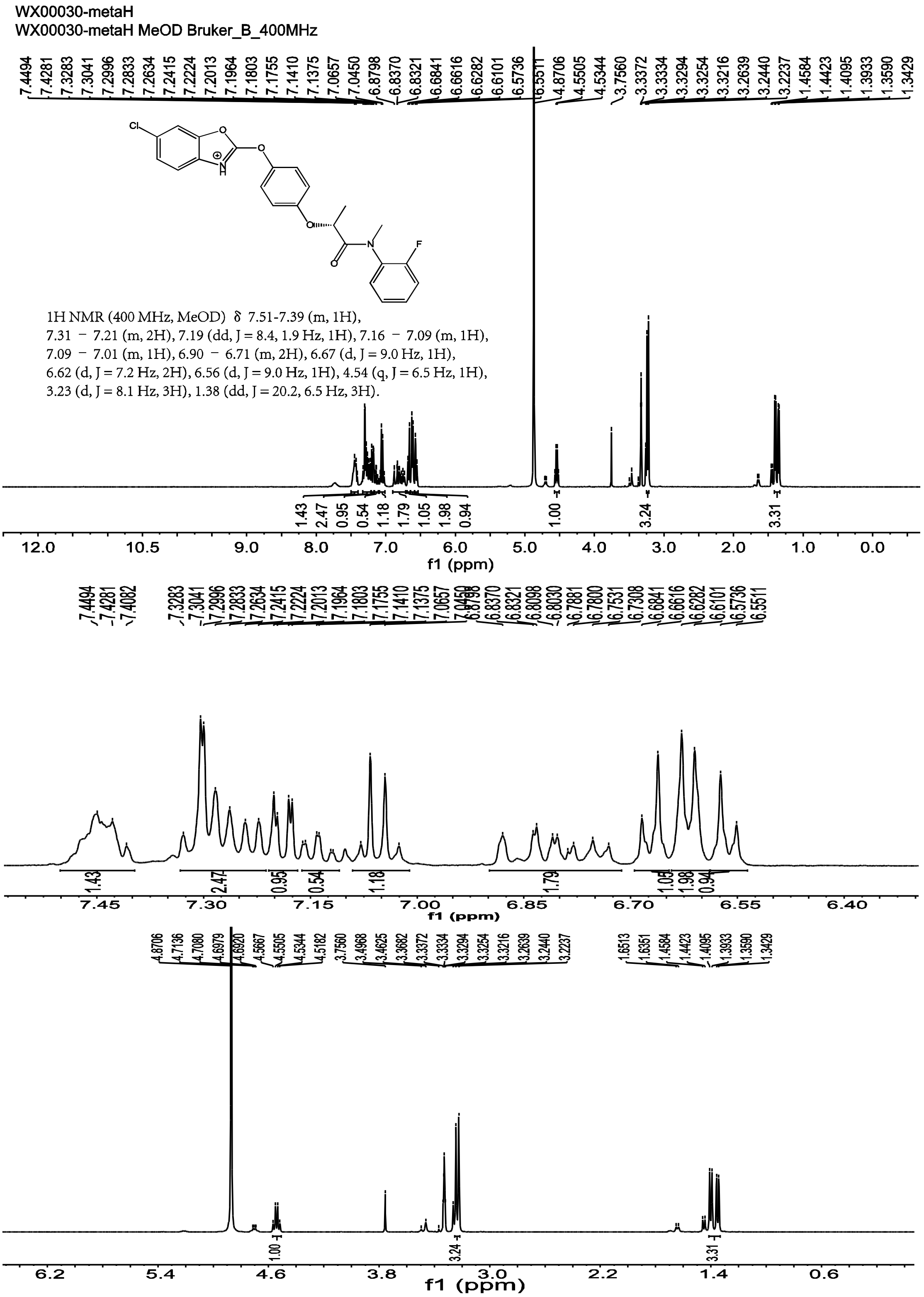

Figure S4. ^{1}H NMR of metamifopH.

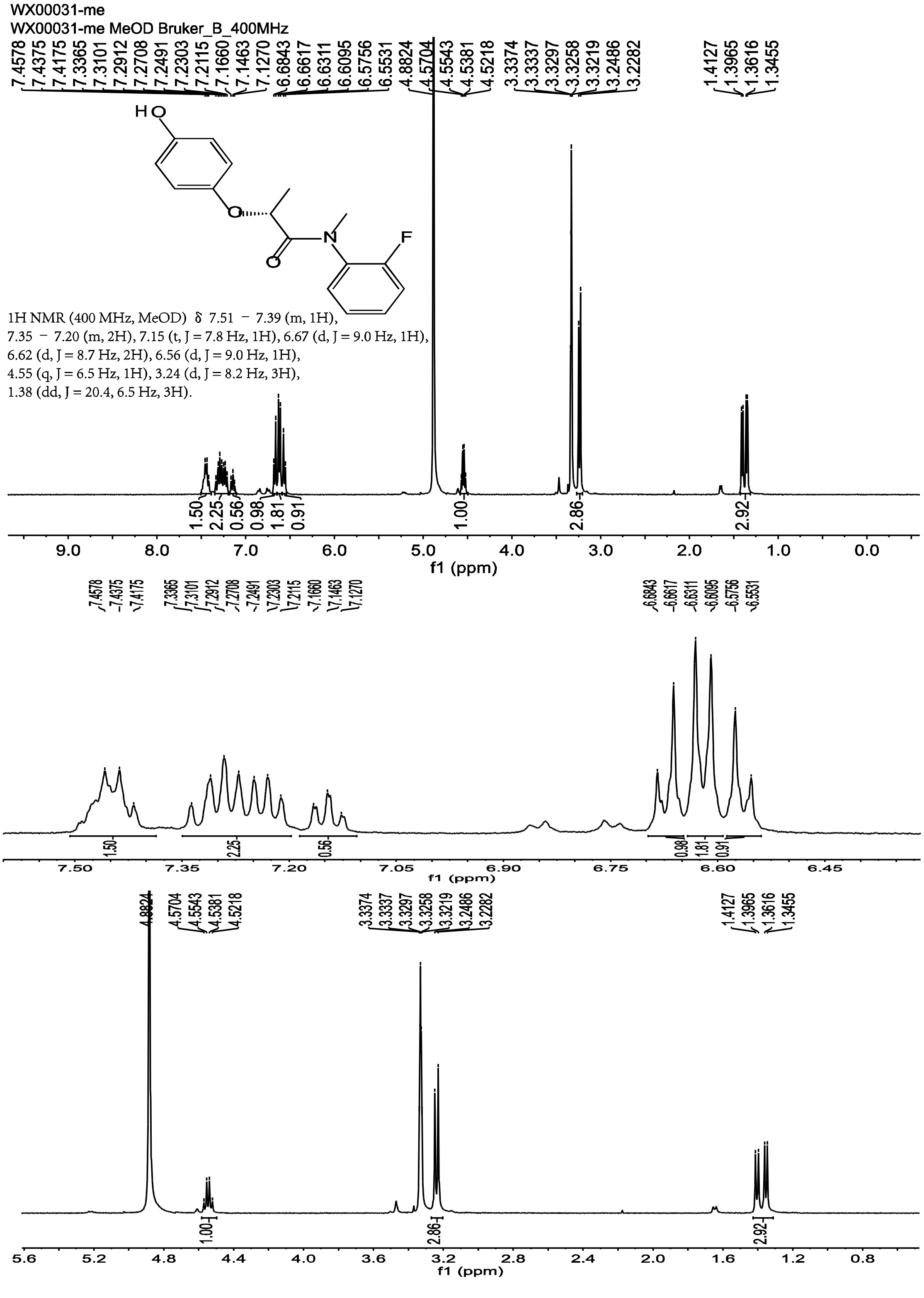

Figure S5. ^{1}H NMR of propanamide.

Methods

Sequencing of chloroplastic ACCase CT domain. Total RNA was isolated from leaves of *Echinochloa crus-galli* using an RNAprep Pure Plant Kit (Tiangen Biotech Co., Ltd., Beijing, China). For RT-PCR, first-strand cDNA was reverse transcribed from the total RNA using the RevertAid RT Kit (Thermo Scientific). Based on the barnyard grass ACCase encoding sequence (GenBank accession HQ395758), a pair of primers (CT4889:5' AAACGCTGCTCTGCTAGGAA 3', CT7342: 5' CTTGCAAACT GTCACTCGGC 3') was designed for PCR amplification of the DNA fragment of the entire CT domain of barnyardgrass chloroplastic ACCase. Each amplification reaction was performed in a total volume of 50 µl with 37.75µl distilled deionized (dd) H_2O, 1 µl of cDNA, 1 µl of each primer (10 µM), 5 µl of 10X *Ex Taq* Buffer, 4 µl of dNTP Mixture, and 0.25 µl of *TaKaRa Ex Taq* DNA Polymerase (250 U/µl). The RT-PCR procedure was as follows: an initial denaturation step at 95 ℃ for 5 min, followed by 35 cycles of denaturation for 30 s at 95 ℃, annealing for 1 min at 57 ℃, extension for 2 min at 72 ℃, and a final elongation step at 72 ℃ for 10 min. The amplification product obtained from at least 6 independent PCR reactions was purified and then sequenced on both strands using gene-specific primers.

Real-time PCR. *Echinochloa crus-galli* was grown to the 2-leaf stage and treated with different doses of metamifop (12.5 and 45 g a.i. ha^{-1}). The aqueous solution with 0.1% Triton-X was used as the untreated control. The aboveground part was harvested at the 2-leaf stage (1 DAT), 3-leaf stage (3 DAT) and 10 days after the flowering (DAF) stage (105 DAT). The level of CT domain gene expression was analyzed by real-time

PCR using β-actin as a reference gene. Triplicate cDNA aliquots for each sample from individual plants were amplified by qPCR. In total, 20 μl of reaction mixture containing 7.4 μl ddH_2O, 1 μl cDNA, 0.8 μl of each primer (10 μM) and 10 μl of SsoFast EvaGreen Supermix (Bio-Rad, USA) was used for qPCR (iQ2 Optical System; Bio-Rad, USA). The qPCR amplification conditions included an initial denaturation at 95 ℃ for 30 s, followed by 40 cycles of 95 ℃ for 5 s and 60 ℃ for 30 s. After amplification, a melting curve analysis from 65 ℃ to 95 ℃ was verified to produce a specific amplified product. A pair of primers (CT4F 5' TTGCCTCTGTTCATCCTT 3', CT4R 5' TCAGCATAACACTCAATTCG 3') for the qPCR amplification of the *acc*D gene was designed, and the primers used for β-actin were as described by Zhibo H. *et al.*[18]. Relative changes in gene expression were calculated by the $2^{-\Delta\Delta Ct}$ method[33].

ACCase activity assay. When plants grew to the 2- to 3-leaf stage, the shoots (3 g per sample) were harvested at the base, quickly frozen in liquid nitrogen, and stored at -80 ℃. The next day, these materials were ground in liquid nitrogen and homogenized in 15 mL of extraction buffer (100 mM Tris-HCl, pH 8.0, 1 mM EDTA, 10% glycerol, 2 mM isoascorbic acid, 0.5% PVP-40, 0.5% PVPP, 20 mM DTT and 1 mM PMSF). The homogenate was then filtered through four layers of muslin and maintained on ice until centrifuged (27 000 g for 15 min at 4 ℃) to remove cell debris. The pellet was discarded, and the supernatant was adjusted using 10% ammonium sulphate and stirred for 30 min. The supernatant was transferred to a new tube and centrifuged at 27 000 g for 30 min. The pellet was discarded. The supernatant was adjusted with 40% ammonium sulphate and stirred for 30 min. The supernatant was centrifuged at 27 000

g for 30 min. The pellet containing the protein extract was resuspended in 2 mL of elution buffer and desalted on a Sephadex G-25 column equilibrated with elution buffer (50 mM Tricine-KOH, pH 8.0, 2.5 mM MgCl2, 50 mM KCl and 1 mM DTT)[34]. The protein extracts were frozen at -80 ℃ and assayed the next day. The total protein contents of the ACCase isolations were determined as described by Bradford[35].

The malonyl-CoA concentration in each sample was determined with an injection volume of 10.0 μl using an HPLC system (Agilent 1200, USA) to record the absorbance peak area at 260 nm. A Thermo Scientific Hypersil ODS-2 (C18) HPLC column (4.6 mm i.d. ×250 mm, 5.0 μm particle size) was used to separate the substances. The separation condition was 5:95 solvent B (MeOH): solvent A (10.0 mM KH_2PO_4, pH 6.7) at a flow rate of 1.0 ml/min. The retention time of malonyl-CoA (Sigma) was 6.27 min. A standard curve was constructed using a series of standard concentrations of malonyl-CoA samples (0.02, 0.04, 0.06, 0.08, 0.1, 0.12 mg/ml) corresponding to the absorbance peak area. The inhibition of barnyard grass ACCase activity by metamifop was determined by the 200 μl volume reaction system for a series of concentrations of metamifop, as described by Zhibo H. *et al.*[18], merely replacing quizalofop-P-ethyl by metamifop. Biological triplicates of the assays were statistically analyzed by ANOVA with Fisher's Protected LSD test. The enzyme activities for the same treatment were averaged when there were no significant differences among the three biological replicates. The data were fitted to the linear model by plotting the probit values of the average ACCase activity inhibition rate against the log dose of metamifop.
